# Supplementary material for: Lymphopenia drives T cell exhaustion in immunodeficient STING gain-of-function mice
Source: EMBO Mol Med. 2025 Aug 13;17(9):2438–61. doi: 10.1038/s44321-025-00292-6 (PMC12423328; doi:10.1038/s44321-025-00292-6)
Supplement: Supplementary file 1 — Appendix [file 44321_2025_292_MOESM1_ESM.pdf]

Appendix

Lymphopenia drives T cell exhaustion in immunodeficient STING gain-of-function mice

Damien Freytag [a], Stéphane Giorgiutti [a,b], Grégoire Hopsomer [a], Nadège Wadier [a], Sabine Depauw [a], Philippe Mertz [a], Fabrice Augé [c], Raphaël Carapito [d], Isabelle Couillin [e], Anne-Sophie Korganow [a,b], Francesca Pala [f], Marita Bosticardo [f], Luigi D. Notarangelo [f], Frédéric Rieux-Laucat [g], Nicolas Riteau [e,h], Peggy Kirstetter [i], Pauline Soulas-Sprauel [a,b,*]

[*] Corresponding author: e-mail address: soulaspa@unistra.fr (P. Soulas-Sprauel); postal address: Inserm U1109, CRBS, 1 rue Eugène Boeckel, F-67000 Strasbourg, France.

This PDF file includes:

Appendix Table S1……………………………………………………………page 2

Appendix Table S2……………………………………………………………page 3

$CD4^+$

Signature details	Size (number of genes)	NES	Nom p-value	FDR q-value
PID_TCR_PATHWAY	64	1.61	0.008	0.050
BIOCARTA_TCR_PATHWAY	44	1.66	0.007	0.032
REACTOME_TCR_SIGNALING	112	1.39	0.027	0.167

$CD8^+$

Signature details	Size (number of genes)	NES	Nom p-value	FDR q-value
PID_TCR_PATHWAY	64	1.34	0.061	0.191
BIOCARTA_TCR_PATHWAY	44	1.39	0.060	0.145
REACTOME_TCR_SIGNALING	112	1.49	0.006	0.079

Appendix Table S1. GSEA signatures.
NES: normalized enrichment score.
Nom p-value: Nominal p-value
FDR q-value: False Discovery Rate q-value

CD4$^+$

Gene	Locus	Control mice – FPKM Mean	STING GOF mice – FPKM Mean	Fold change (STING GOF/control)	p value	q value
cd3e	chr9:44998742-45009590	443.883	684.158	0.624149	0.00005	0.000397557
il7r	chr15:9506158-9529876	189.951	65.0465	-1.54608	0.00005	0.000397557
cdkn1b	chr6:134920400-134925525	108.714	89.3016	-0.283779	0.025	0.0789669
bcl2	chr1:106538178-106714290	44.0479	51.3457	0.22117	0.1275	0.269564
gfi1	chr5:107716654-107725805	8.02892	17.9484	1.16058	0.00005	0.000397557
socs1	chr16:10783808-10785536	17.496	32.3648	0.887397	0.00015	0.00107805

CD8$^+$

Gene	Locus	Control mice – FPKM Mean	STING GOF mice – FPKM Mean	Fold change (STING GOF/control)	p value	q value
cd3e	chr9:44998742-45009590	491.595	578.488	0.234817	0.04835	0.136132
il7r	chr15:9506158-9529876	253.798	124.345	-1.02933	0.00005	0.000423756
cdkn1b	chr6:134920400-134925525	86.0948	65.1919	-0.401234	0.0014	0.00782091
bcl2	chr1:106538178-106714290	51.4897	80.2689	0.640556	0.00005	0.000423756
gfi1	chr5:107716654-107725805	6.8492	12.5575	0.874541	0.0001	0.000799047
socs1	chr16:10783808-10785536	18.4175	32.9478	0.839101	0.0001	0.000799047

Appendix Table S2. Data of RNA-seq analysis for selected genes.
Data are represented as the mean for FPKM for each group of mice, with the results of the statistical test.
