# Supplementary material for: Lymphopenia drives T cell exhaustion in immunodeficient STING gain-of-function mice
Source: EMBO Mol Med. 2025 Aug 13;17(9):2438–61. doi: 10.1038/s44321-025-00292-6 (PMC12423328; doi:10.1038/s44321-025-00292-6)
Supplement: Supplementary file 5 — Expanded View Figures [file 44321_2025_292_MOESM5_ESM.pdf]

Expanded View Figures

Figure EV1. T cell exhaustion phenotype in STING GOF mice is acquired during the transition from naive to memory T cells.

(**A**, **B**) Immunophenotyping of splenic T cells from STING GOF mice and their WT littermate controls by flow cytometry. Proportion of PD-1-, TIGIT-, TIM-3- and LAG-3-expressing cells among (**A**) total $CD4^+$ or $CD8^+$ T cells and (**B**) naive ($CD44^{low}CD62L^+$), central memory (Cen mem, $CD44^{high}CD62L^+$) and effector memory (Eff mem, $CD44^+CD62L^-$) among $CD4^+$ or $CD8^+$ T cells, from STING GOF mice and their WT littermate controls. Each data point corresponds to one mouse; mean ± SEM are shown per population for four mice from two independent experiments (biological replicates). Statistical significances are calculated with a two-tailed Mann–Whitney test; ns (non-significant), $P > 0.05$.

 © The Author(s)

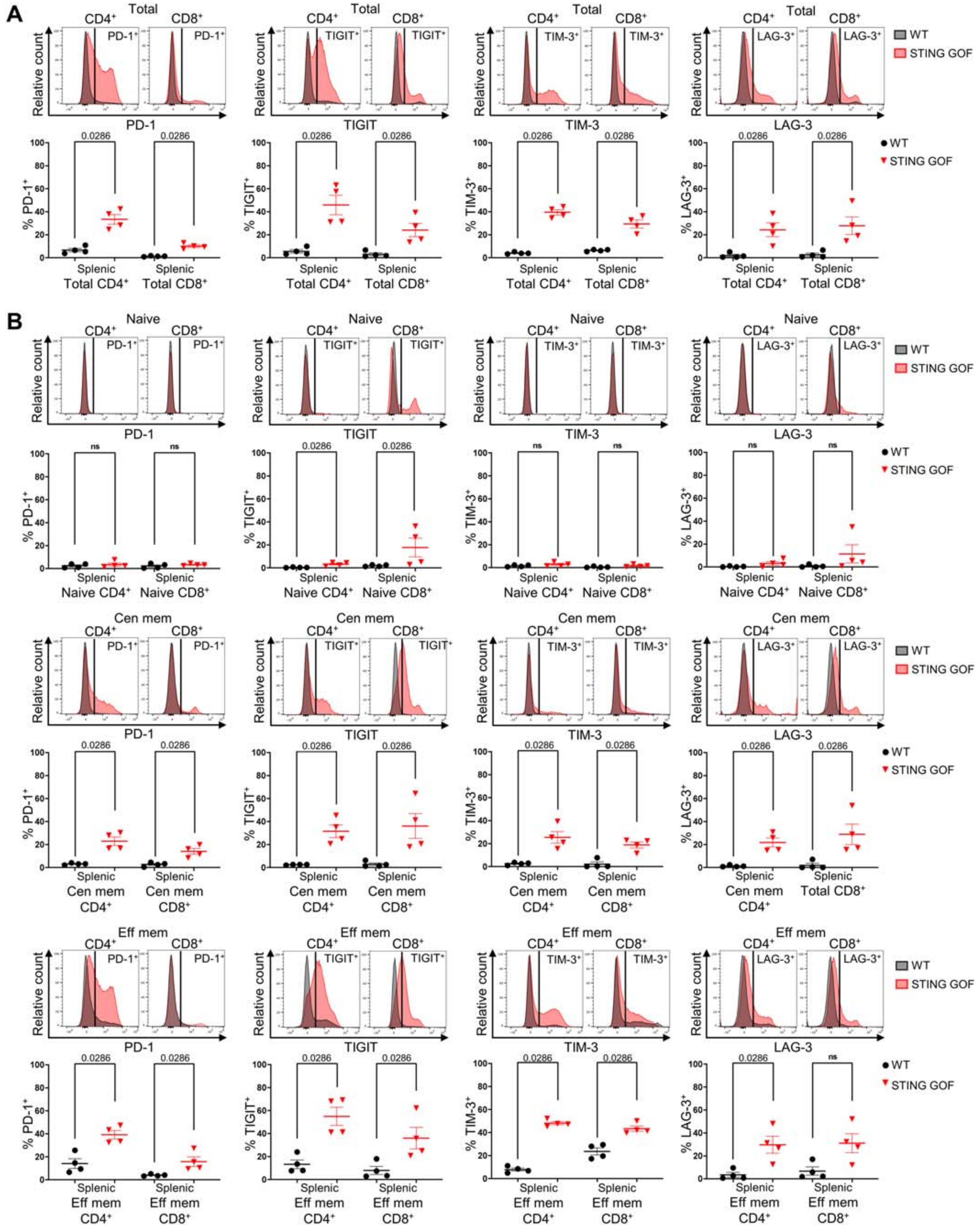
A
Total
CD4+
CD8+
PD-1+
TIGIT+
TIM-3+
LAG-3+
Relative count
PD-1
TIGIT
TIM-3
LAG-3
WT
STING GOF
0.0286
% PD-1+
% TIGIT+
% TIM-3+
% LAG-3+
Splenic Total CD4+
Splenic Total CD8+
B
Naive
ns
Splenic Naive CD4+
Splenic Naive CD8+
Cen mem
Splenic Cen mem CD4+
Splenic Cen mem CD8+
Splenic Total CD8+
Eff mem
Splenic Eff mem CD4+
Splenic Eff mem CD8+

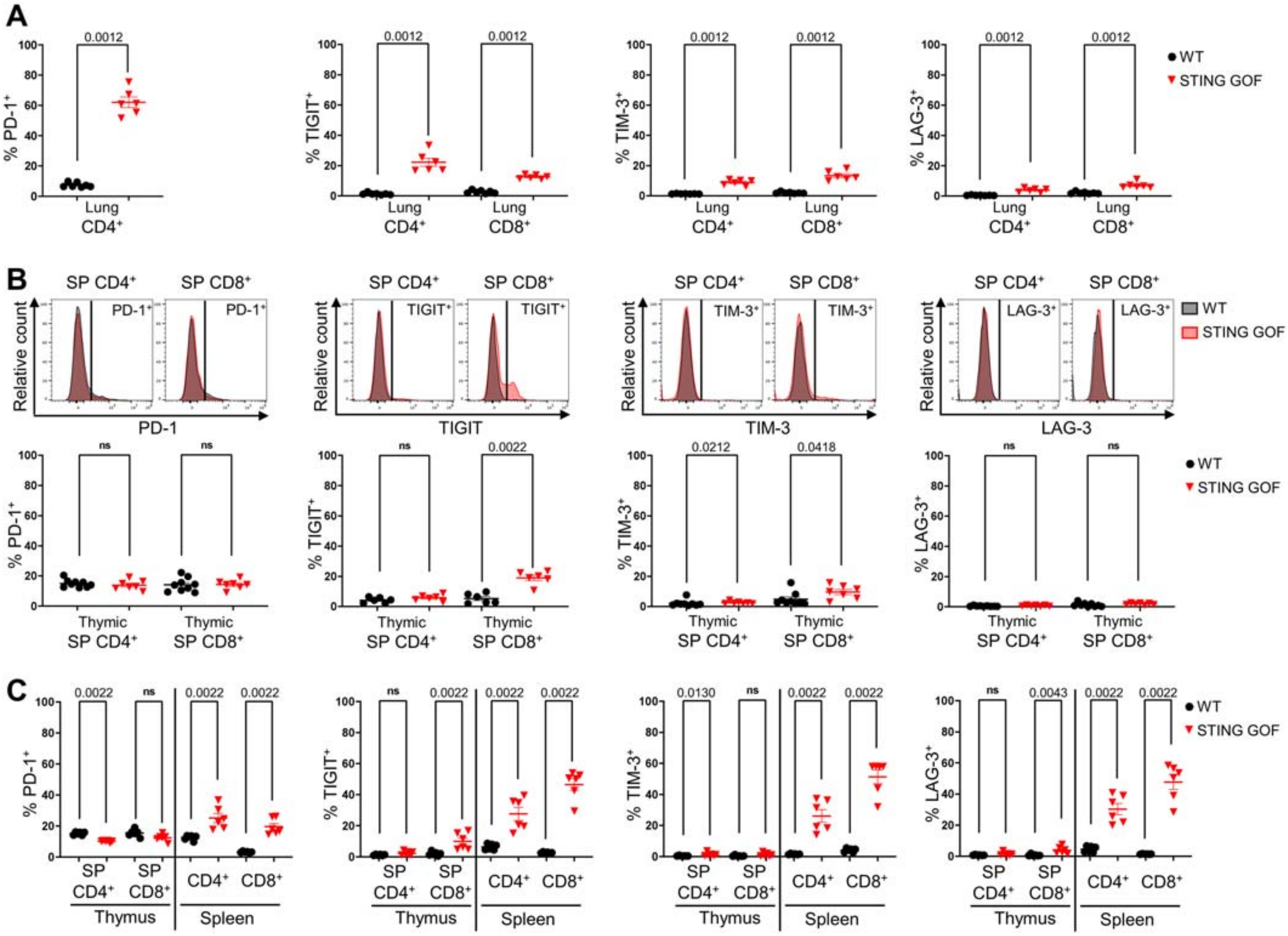

Figure EV2. T cell exhaustion in STING GOF mice is acquired early in life and in the peripheral environment.

(**A**) Proportion of PD-1-, TIGIT-, TIM-3-, and LAG-3-expressing cells among lung CD4+ or CD8+ T cells from STING GOF mice and their WT littermate controls. (**B**) Proportion of PD-1-, TIGIT-, TIM-3-, and LAG-3-expressing cells among thymic CD4+ or CD8+ SP T cells from STING GOF mice and their WT littermate controls. Representative histograms are shown. (**C**) Proportion of PD-1-, TIGIT-, TIM-3-, and LAG-3-expressing cells among thymic and splenic CD4+ or CD8+ SP T cells from 2-week-old STING GOF mice and their WT littermate controls. Each data point corresponds to one mouse; mean ± SEM are shown per population for six to nine mice from two (**A**) and three (**B**, **C**) independent experiments (biological replicates). Statistical significances are calculated with a two-tailed Mann–Whitney test; ns (non-significant), $P > 0.05$.

 © The Author(s)

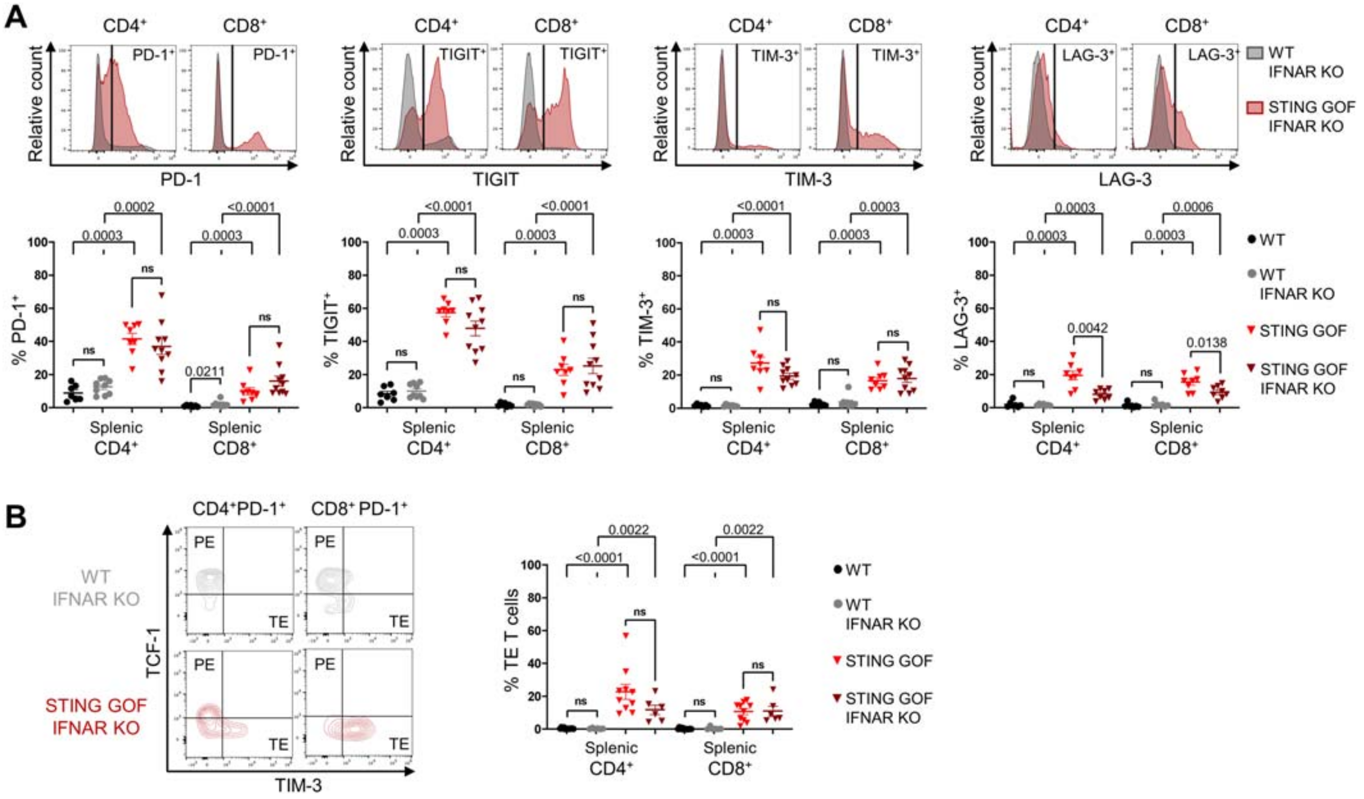

Figure EV3. T cell exhaustion in STING GOF mice is independent of type I IFNs.

(**A**) Proportion of PD-1-, TIGIT-, TIM-3-, and LAG-3-expressing cells among splenic CD4+ or CD8 + T cells from STING GOF IFNAR KO mice and their respective WT littermate controls. Data were compared with the previous result obtained from STING GOF mice and their WT littermate controls (Fig. 1). Representative histograms are shown for the IFNAR KO pair. (**B**) Proportion of terminally exhausted (TE) T cells among splenic CD4⁺ or CD8⁺ T cells from STING GOF IFNAR KO mice and their respective WT littermate controls. Data were compared with results obtained from STING GOF mice and their WT littermate controls (Fig. 1). Representative contour plots are shown for the IFNAR KO pair. Each data point corresponds to one mouse; mean ± SEM are shown per population for six to ten mice from at least three independent experiments (biological replicates). Statistical significances are calculated between IFNAR KO groups as well as between STING GOF groups with a two-tailed Mann–Whitney test.

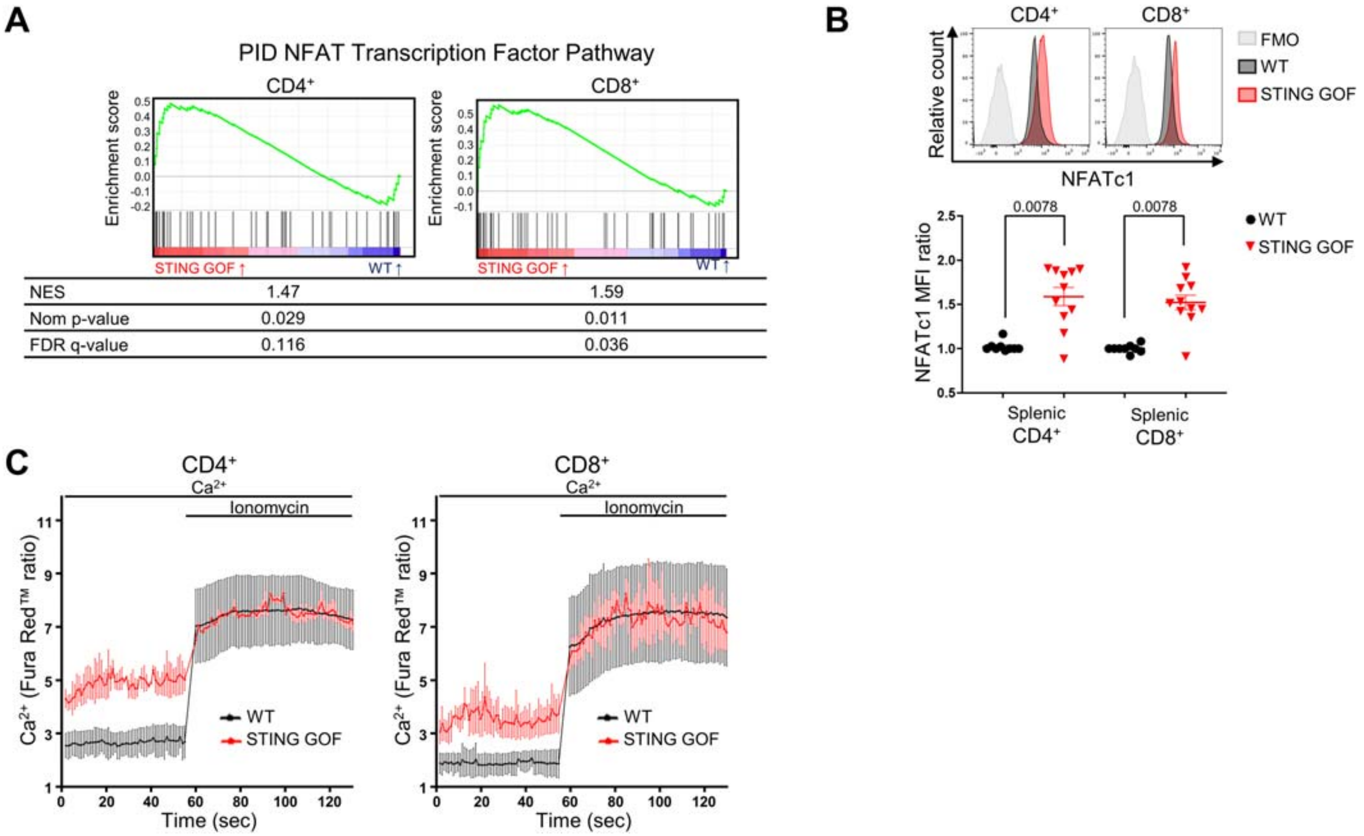

Figure EV4. **Ca^{2+}-NFAT activation in STING GOF T cells.**

(**A**) GSEA of the NFAT pathway signature (PID) among genes deregulated in STING GOF versus WT CD4+ or CD8+ T cells. Enrichment plots, normalized enrichment score (NES) and nominal *p* value (*p* value) are shown for each analysis. (**B**, **C**) Immunophenotyping and Ca^{2+}levels of splenic T cells from STING GOF mice and their WT littermate controls by flow cytometry. (**B**) Ratio of total NFATc1 mean fluorescence intensity (MFI) in splenic CD4+ or CD8+ T cells from STING GOF mice and their WT littermate controls. Ratio was normalized on the WT control of each analysis. Representative histograms are shown. Each data point corresponds to one mouse; mean ± SEM are shown per population for nine to eleven mice from seven independent experiments (biological replicates). Statistical significances are calculated with the Wilcoxon signed-rank test with a hypothetical value of 1: $^{**}P < 0.01$. (**C**) Relative cytosolic Ca^{2+} levels monitored by Fura Red™ ratio in splenic CD4+ or CD8+ T cells from STING GOF mice and their WT littermate controls. Splenocytes were recorded in DMEM containing 1.8 mM Ca^{2+} and stimulated with 1 µg/mL ionomycin after 50 s as a positive control. Fura Red™ intensities in VL4 (405 nm-excitation; 660 nm-emission) and BL3 (488 nm-excitation; 695 nm-emission) channels were recorded, corresponding to Ca^{2+}-bound and Ca^{2+}-free Fura Red™, respectively. Ratio (VL4/BL3) of these Fura Red™ MFI according to time are plotted in a curve graph where each data point represents the mean of two independent experiments (biological replicates), and their error bars the SEM.

 © The Author(s)

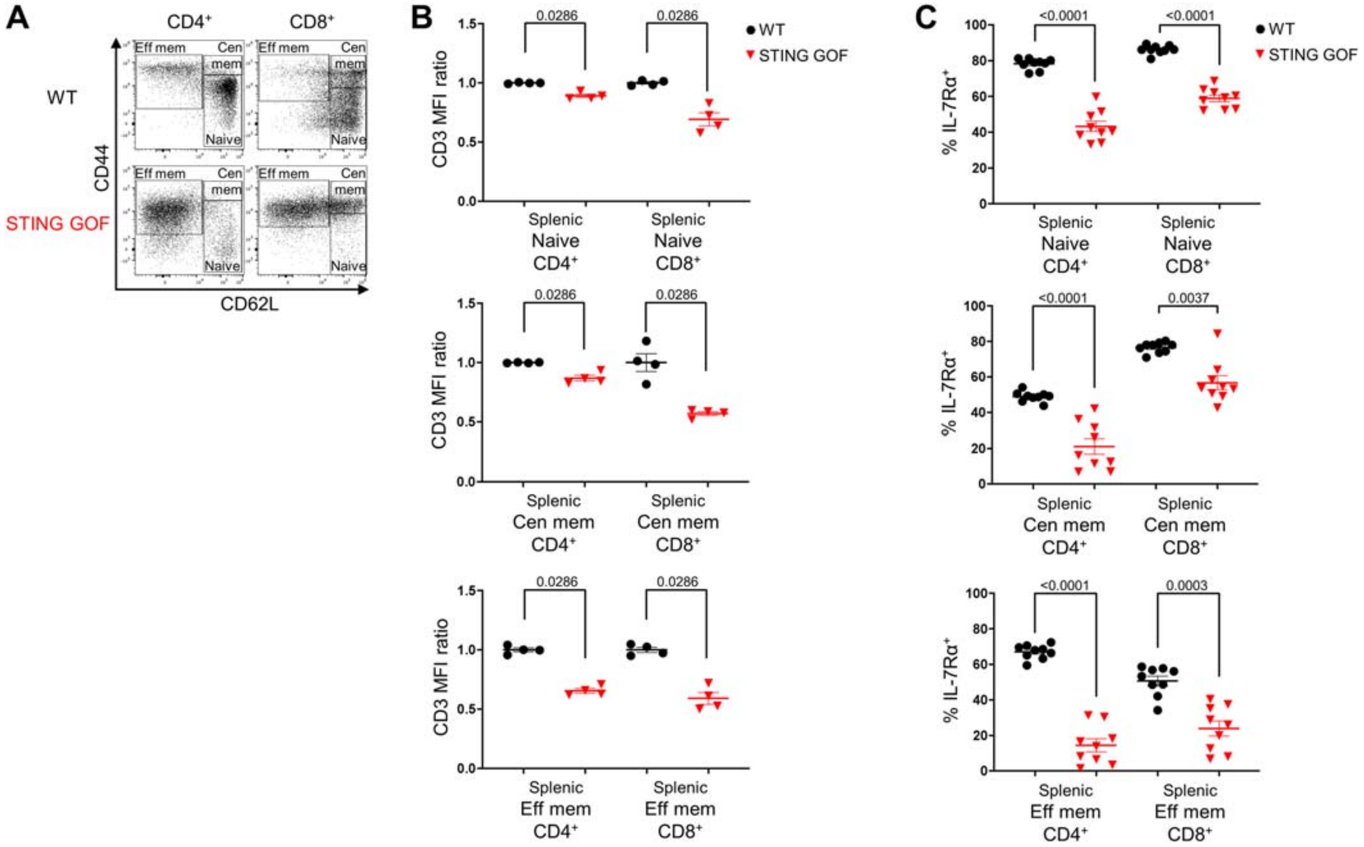

Figure EV5. TCR and IL-7R engagement since the naive T cell stage in STING GOF mice.

(**A**, **C**) Immunophenotyping of splenic T cells from STING GOF mice and their WT littermate controls by flow cytometry. (**A**) Representative gating strategy of naive ($CD44^{low}CD62L^{+}$), central memory (Cen mem, $CD44^{high}CD62L^{+}$) and effector memory (Eff mem, $CD44^{+}CD62L^{-}$) for both $CD4^{+}$ and $CD8^{+}$ T cells from STING GOF mice and their WT littermate controls. (**B**) Ratio of CD3 mean fluorescence intensity (MFI) on splenic naive, central memory and effector memory $CD4^{+}$ or $CD8^{+}$ T cells from STING GOF mice and their WT littermate controls. Ratio was normalized on the mean of WT controls of each analysis. (**C**) Proportion of IL-7Rα-expressing cells among splenic naive, central memory and effector memory $CD4^{+}$ or $CD8^{+}$ T cells from STING GOF mice and their WT littermate controls. Each data point corresponds to one mouse; mean ± SEM are shown per population for four (**B**) to nine (**C**) mice from two (**B**) and three (**C**) independent experiments (biological replicates). Statistical significances are calculated with a two-tailed Mann–Whitney test.

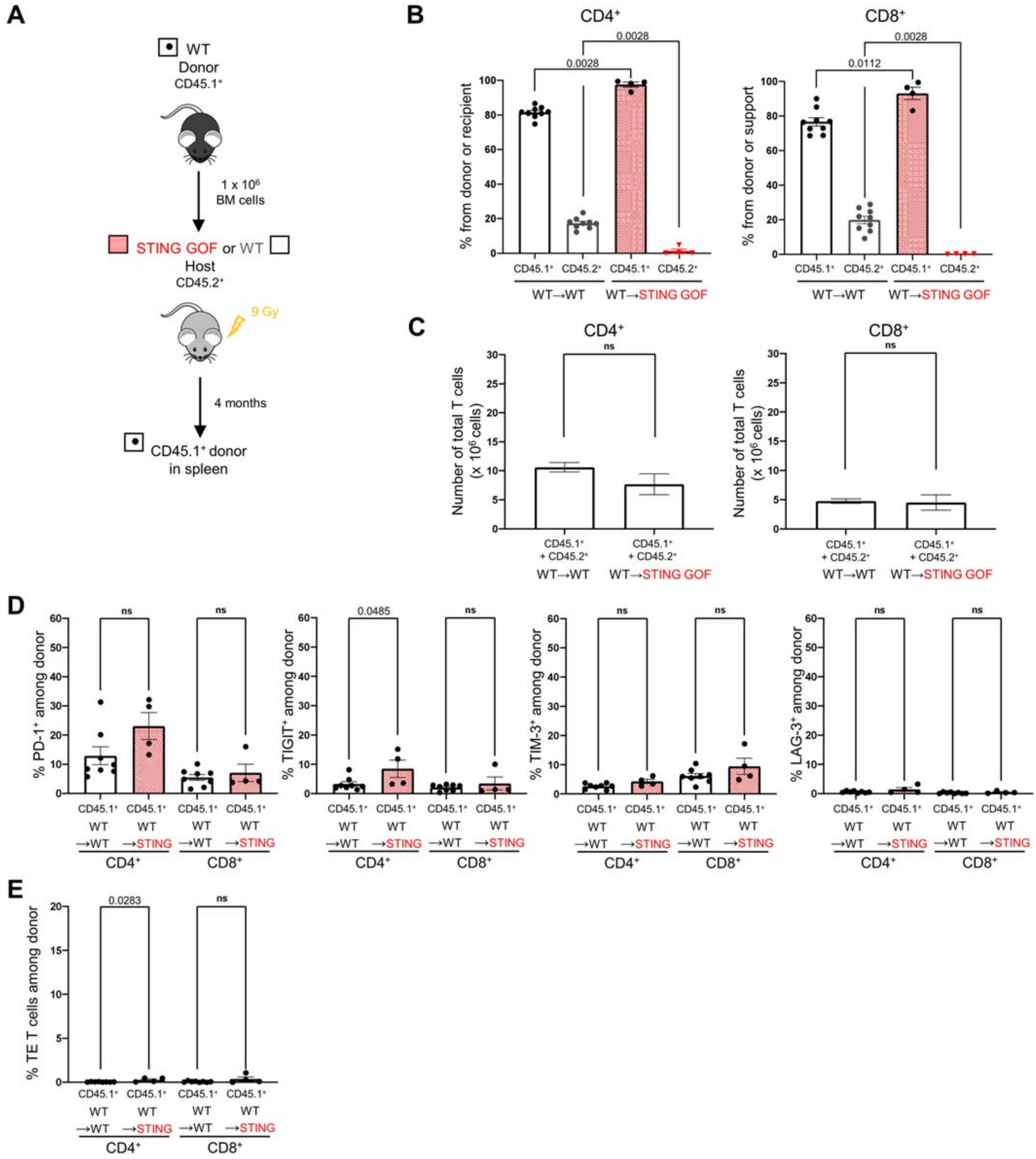
A
WT Donor CD45.1+
1 x 10^6 BM cells
STING GOF or WT Host CD45.2+
9 Gy
4 months
CD45.1+ donor in spleen
B
CD4+
CD8+
% from donor or recipient
% from donor or support
0.0028
0.0112
CD45.1+ CD45.2+ CD45.1+ CD45.2+
WT→WT WT→STING GOF
C
Number of total T cells (x 10^6 cells)
ns
CD45.1+ + CD45.2+
WT→WT WT→STING GOF
D
% PD-1+ among donor
% TIGIT+ among donor
% TIM-3+ among donor
% LAG-3+ among donor
0.0485
ns
CD45.1+ WT →WT
CD45.1+ WT →STING
CD4+ CD8+
E
% TE T cells among donor
0.0283

Figure EV6. STING GOF radioresistant stroma is not sufficient to induce T cell exhaustion.

(**A–E**) STING GOF mice and their WT littermate controls ($CD45.2^+$) were lethally irradiated and then reconstituted with WT BM donor cells ($CD45.1^+$) to generate WT→STING GOF and WT→WT mice. Four months later, spleens were assessed for T cell reconstitution and immunophenotyping. (**A**) Strategy of WT BM cells transplantations into STING GOF or WT irradiated recipient mice. (**B**) Proportion of cells derived from WT BM donor cells ($CD45.1^+$) or host cells ($CD45.2^+$) among splenic $CD4^+$ or $CD8^+$ T cells from WT→STING GOF and WT→WT mice. (**C**) Absolute numbers of total ($CD45.1^+$ and $CD45.2^+$) splenic $CD4^+$ or $CD8^+$ T cells from WT→STING GOF and WT→WT mice. (**D**) Proportion of PD-1-, TIGIT-, TIM-3-, and LAG-3-expressing cells among splenic $CD4^+$ or $CD8^+$ T cells derived from WT BM donor cells ($CD45.1^+$) from WT→STING GOF and WT→WT mice. (**E**) Proportion of terminally exhausted (TE) T cells among splenic $CD4^+$ or $CD8^+$ T cells derived from WT BM donor cells ($CD45.1^+$) from WT→STING GOF and WT→WT mice. Each data point corresponds to one mouse; mean ± SEM are shown per population for four to nine mice from two independent experiments (biological replicates). Statistical significances are calculated with a two-tailed Mann–Whitney test; ns (non-significant), $P > 0.05$.

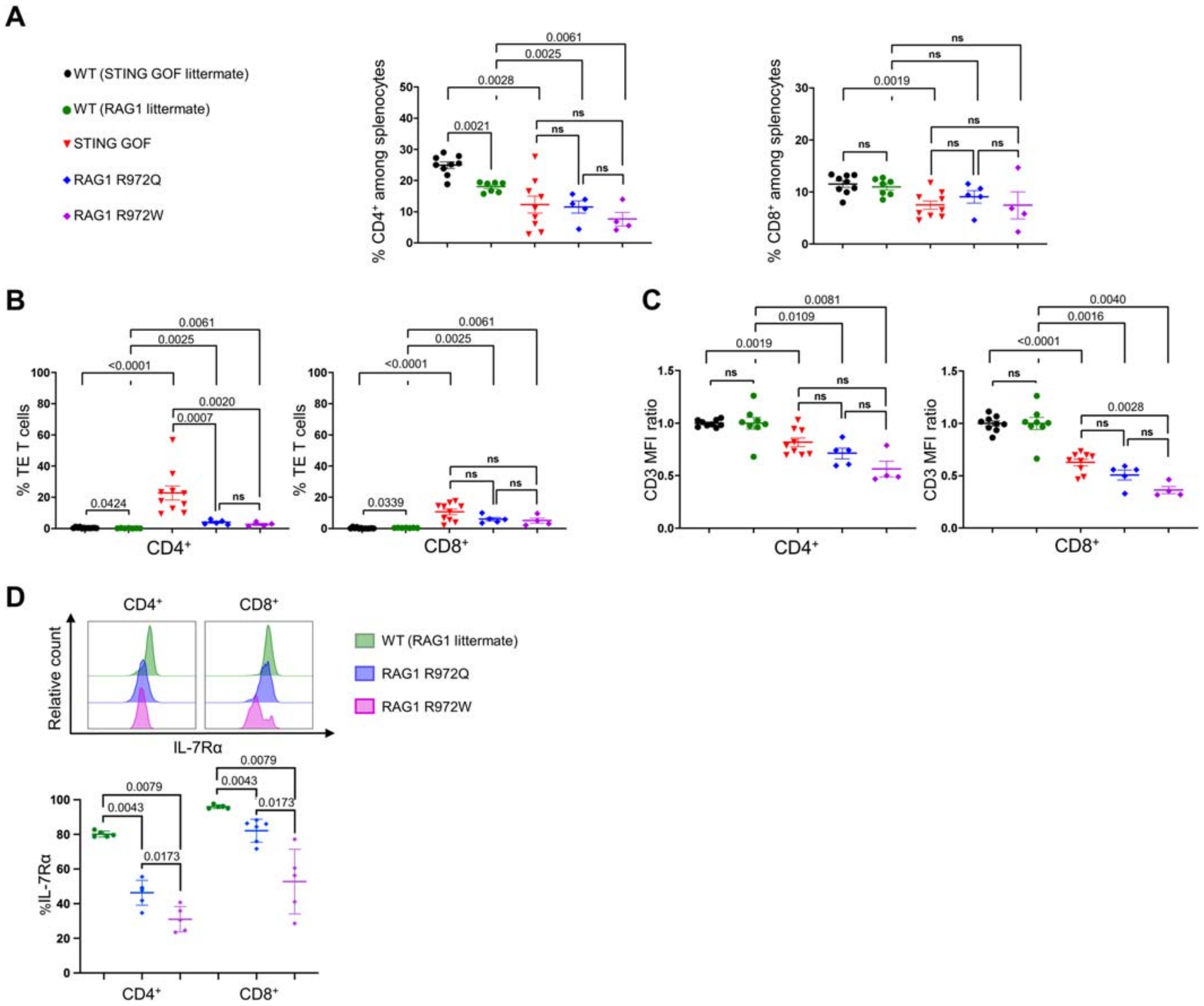

Figure EV7. Mice carrying *Rag1* hypomorphic mutations also display lymphopenia-associated T cell exhaustion.

(**A–C**) Immunophenotyping of T cells from the spleen of hypomorphic RAG1 R972Q and R972W mice and their WT littermate controls by flow cytometry. Data were compared with the previous result obtained from STING GOF mice and their WT littermate controls (Figs. 1F and 3B). (**A**) Proportion of splenic $CD4^+$ or $CD8^+$ T cells from hypomorphic RAG1 mice and their WT littermate controls. (**B**) Proportion of terminally exhausted (TE) T cells among splenic $CD4^+$ or $CD8^+$ T cells from hypomorphic RAG1 mice and their WT littermate controls. (**C**) Ratio of CD3 mean fluorescence intensity (MFI) on splenic $CD4^+$ or $CD8^+$ T cells from hypomorphic RAG1 mice and their WT littermate controls. Ratio was normalized on the mean of WT controls of each analysis. (**D**) Proportion of IL-7Rα-expressing cells among splenic total CD4+ or CD8 + T cells from hypomorphic RAG1 mice and their WT littermate controls. Representative histograms are shown. Each data point corresponds to one mouse; mean ± SEM are shown per population for four to ten mice from two independent experiments (biological replicates). Statistical significances are calculated with a two-tailed Mann–Whitney test; ns (non-significant), $P > 0.05$.

 © The Author(s)
